# Medical Opinion and Movement

**Published:** 1911-07-22

**Authors:** 


### MEDICAL OPINION AND MOVEMENT.

### Pneumonia in Infants.

Dr. Engel draws attention to the fact that there is an important mechanical factor in the predisposition of infants to pneumonia. This mechanical factor consists in compression of the lung owing to the volume of the infantile lungs being too great in relation to the thorax. In consequence, the ribs make deep impressions on the lung tissue. In order to demonstrate the matter, longitudinal sections of the lung tissue must be made. In the right lung the initial pneumonic foci occur in the subpleural pulmonary tissue of the upper lobe, immediately subjacent to the costal impression. They enlarge and extend along the surface of the lung towards the base. In the left lung they are localised at the commencement in the inferior lobe, immediately behind the dorsal aspect of the heart. The initial foci of pneumonia, therefore, are localised at those portions of the lungs which are subjected to some amount of compression and are, in consequence, the least efficiently ventilated. Similar observations have also been made by Dr. Tendeloo.

### Salicylates and Alkalies.

About six years ago Dr. Frey showed by experiments on animals, and also on himself, that albuminuria and casts, as a result of the administration of salicylates, did not occur except when the urine was acid. It appeared, therefore, from these observations that the renal irritation was not due to a general intoxication by the salicylates, but was a purely local effect caused by the salicylate set free in an acid urine. Dr. Ehrmann has contested this view, as he has found that in dogs administration of considerable doses of bicarbonate of soda, sufficient to diminish the acidity of the urine, frequently caused albuminuria, without the intervention of salicylic acid. Dr. Glässgen has recently investigated the subject anew by observations on patients in the Strassburg clinic. In the first place he finds that the continuous use of salicylic preparations does undoubtedly give rise to albuminuria. Further, according to his observations, the administration of 10 grammes per day of sodium bicarbonate causes the complete disappearance of this albuminuria, which however returns as soon as the alkaline medication is omitted. As a general rule, Dr. Glässgen gives twice as much alkali as salicylates, but does not exceed 10 grammes a day. According to his experience, 6 to 10 grammes of bicarbonate of soda produce no undesirable symptoms and do not in any way diminish the therapeutic effects of the salicylates. They are especially indicated as a prophylactic measure in cases where the kidneys are already affected.

### Composition of Rachitic Bones.

It is generally recognised that rachitic bones contain less calcium than normal bones. Dr. Gassmann has investigated the composition of rachitic bones in regard to other elements to determine whether any variations in them are determining factors in the condition. An account of his research appears in the *Zeitschrift für Physiologische Chemie*. He finds that normal bones contain about 1 per cent. more water than rachitic bones, this difference being fairly proportional to that of the calcium. The quantities of phosphoric and carbonic acid are also diminished, but the proportions of the acids to the calcium are the same as in normal bones, so that there appears to be the same stable combination of calcium with these acids in rachitic as in normal bones. Magnesia is present in greater proportions in rachitic than in normal bone. In connection with this fact it is interesting to note that the human teeth at the present time contain more magnesia than those of the prehistoric period, and they are at the same time less resistant. Further, teeth predisposed to caries are richer in magnesia. It would appear, therefore, that this augmentation in magnesia is an important factor. The proportions of chlorine, sodium, potassium, and nitrogen do not show any modification.

### The Recto-sigmoidoscope.

In an article in the *Berliner Klinische Wochenschrift* on the utility of the recto-sigmoidoscope in the diagnosis of affections of the lower bowel, Dr. Ewald draws attention to one particular condition, which is not generally recognised and which can only be properly diagnosed by local inspection with this instrument. It consists in a varicosity of the superior hæmorrhoidal plexus, giving rise to persistent hæmorrhage, though generally small in amount, the persistency, however, often causing severe anæmia, with other attendant constitutional symptoms. In such a case the hæmatologic tableau presents the usual characters of a chlorosis from hæmorrhage. Examination of the fæces shows the presence of blood, and the patient usually suffers from chronic constipation. The patients are generally comparatively young—twenty-five to forty —and owing to the anæmia they complain of intense lassitude, anorexia, headache and sometimes vertigo, insomnia, and palpitation. The hands and feet are cold, but the least effort causes perspiration. Ordinary rectal examination is negative. External and internal hæmorrhoids are either slight or altogether absent, and they do not give rise to any hæmorrhage. Inspection with the recto-sigmoidoscope shows the presence of varicose nodules about the size of lentils, sometimes larger, 10 or 15 centimetres above the anus and sometimes higher still. These nodules look like little purplish buttons raised above the surrounding mucous membrane, which is rather pale. They easily bleed on touching them with an instrument. For treatment the author advises the application of the thermo-cautery, and subsequent rectal injections of 3 to 5 per cent. solution of perchloride of iron. Later on he advises small injections of 200 to 250 grammes of oil and bismuth. The condition should be cured in the course of four or five weeks.

### Demonstration of Tubercle Bacilli.

Hart and Lessing in No. 9, and Bachrach and Necker in No. 12, of the *Wiener Klin. Wochenschrift*, report use of antiformin for the above purpose, as this reagent dissolves extraneous organisms without—in a 15 per cent. solution—affecting the vitality of the bacillus of Koch. The former authors recommend systematic investigation of the lymphatic system in children by making fine serial sections and examining microscopically in the usual way, and then homogenising them with antiformin and inoculating animals with the sediment. It is claimed that thus even histologically unaltered tissues may be shown to harbour tubercle bacilli. It will be recalled that the modern French school does not consider the classical microscopic appearances of tubercle a *sine qua non* in diagnosis. Bachrach and Necker examined thirty-seven specimens of urine in the following manner: Injection of the sediment into the inguinal region of guinea-pigs after previous bruising of the glands, removal of the glands after ten to eleven days, treatment of them with 15 per cent. antiformin solution for five hours; the sediment after centrifying being then stained in the usual way. Twenty-five of the specimens gave a positive result, and it is stated that the remaining twelve showed the cases later to be non-tuberculous. The investigation was controlled by peritoneal inoculation, and the agreement of results was exact. As even stronger solutions of antiformin are harmless to tubercle bacilli but fatal to virulent pus organisms, it is claimed that the addition of this reagent before inoculation protects the animals used in experiments from dying from septic infection, while the saving in time is obvious.

### The Bacteriology of Leprosy.

In the *Indian Medical Gazette* a special supplement is devoted to the bacteriological researches of Capt. T. S. B. Williams, who believes that the micro-organism of this disease is a highly pleomorphic one, some of whose manifestations have not hitherto been recognised. He says that leprosy may be caused by perhaps several strains of a streptothrix, which may be: (1) a non-acid-fast streptothrix with interlacing filaments; (2) a non-acid-fast diphtheroid bacillus, capable of becoming acid-fast under certain defined conditions; (3) a definitely acid-fast filamentous streptothrix; and (4) an acid-fast bacillus which is the broken-down stage of a streptothrix. Some of these forms he has been able to cultivate; after which, naturally enough, the possibilities of vaccine therapy are considered. At present the author is unwilling to claim any definite cures by the use of vaccines made from his cultures, or to arouse too high an expectation of cure. His results are still in an experimental stage, and he is by no means prepared to regard his present vaccine as necessarily the best way of utilising the organism. But definite reactions have been secured in leprous patients, and at least Captain Williams claims that his vaccines control the progress of the disease and prevent the outbreak of new lesions. Some anæsthetic patients have recovered sensation under vaccine treatment; and ulcers, nodules, tubercles, and other lepromata often clear up wholly or partly. In early cases he thinks there is a good chance of securing entire cure. The article is illustrated by numerous illustrations in colours, excellently reproduced.

### A New Use for Separated Milk.

It should be a matter of some hygienic importance and for congratulation that a new use for separated milk has been found in England. A patent process is now being employed commercially to make use of this product and to convert it into a hard tough substance like ivory in texture and colour. The new material is said to be readily worked on the lathe and capable of being planed, embossed, or moulded. Fortunately it possesses the great advantage over celluloid and other compositions in being non-inflammable. Some years ago the new composition was in demand for the manufacture of Murphy buttons, since when specially prepared it is digestible. At Prague and at Leipzig such buttons are still used, but they seem to be unknown in this country. The substance seems capable of an extended application, and its use might certainly be encouraged if only for the reason that this development may lead to a decrease in the use of separated milk for the production of certain brands of tinned milk, the pernicious effect of which, when used as food for infants, are only too well known to the profession.

### The Prophylaxis of Ophthalmia Neonatorum.

A tendency has been noticeable for some time among obstetricians to abandon the strict Crédé method of preventing ophthalmia neonatorum. Some rely simply on careful wiping of the eyes immediately the head has passed the perineum, either with plain boiled water or with boracic acid solution; others employ also argyrol, protargol, or other antiseptics less irritating than nitrate of silver. Dr. Otto v. Herff contributes to the *Münchener Med. Wochenschrift* an account of a silver preparation named sophol, for which he claims many advantages. This compound is an organic silver salt containing about 20 per cent. of silver. It has the property of stability to a much greater degree than have most silver compounds. It causes less irritation of the conjunctiva than any other preparation except argyrol, with which it stands on an equality in this respect. Argyrol has also been used by Dr. v. Herff with good results, but he has abandoned it on account of the want of stability in the composition of the solution. The author allows two drops of the sophol to fall on the closed eyelids near the inner canthus. Thus the edges of the lashes and lids are well saturated, the localities where gonococci are especially prone to lurk. The child opens its eyes itself; or if not, the midwife draws the lids gently apart. Nothing further is necessary except gently to wipe the eyes clean. By this method 7,500 children have been treated in Basle; of these about 1,100 are suspected to have been exposed to the gonococcus during birth. There have been only two infections amongst these children, one early and one late. The figures speak for themselves. The author is favourably impressed with sophol for curing ophthalmia which has already arisen, but is not yet willing to commit himself definitely on this point.

### Corneal Opacity due to Lead.

An ingenious and novel treatment for the dense white corneal opacity which follows the treatment of ulceration of the cornea by lead salts is recorded in the *Ophthalmoscope* by Major Elliott. The patient, a Hindu woman, had treated herself for an inflamed eye with three successive applications of sugar of lead dissolved in rose water. The inflammation duly subsided, but a dense white plaque remained on the cornea, for which she wanted relief on æsthetic grounds. The author scraped the ulcer freely, but without materially affecting the opacity. He then irrigated the eye with a weak aqueous solution of sulphuretted hydrogen, when the white patch at once turned brown. Although the cornea remained just as opaque as before, the opacity was now no longer obvious to every beholder, though still quite evident on close examination. The patient was so satisfied with this result that she declined all further treatment and has not since been seen. Major Elliott is not aware that anyone has forestalled this resourceful utilisation of the elementary chemistry learnt in the early stages of the medical curriculum, but thinks it possible that it is so. At any rate, the dodge is one worth remembering, though it might not be so successful for a European as for an Indian patient.

### On Boils.

In the light of a personal and prolonged experience of recurrent furunculosis, Dr. Shingleton Smith contributes some valuable observations on this subject to the *Bristol Medico-Chirurgical Journal*. For several months he suffered from very severe boils, each one appearing as the previous one was healing, which caused great pain, enlargement of the inguinal glands, and partial disablement. The suction cups known as Bier's treatment were tried repeatedly and in all stages of evolution of the boils; but nearly always they made the condition worse instead of better, except in the case of large boils which had already been freely incised. The cups also caused aggravation of the severe pain of the boils. Other treatments were equally inutile—sulphur and mercurial inunctions, tonics, hot disinfectant baths. At last Dr. Smith had a culture taken and a vaccine made; the organism found was the staphylococcus pyogenes aureus. He began with twenty million for a dose, and a healing boil promptly relapsed and became painful: next day the temperature rose to 103° F. Amelioration then set in, and increasing doses once a week soon controlled the furunculosis, though slight local and constitutional evidences of a negative phase appeared at first. In six months the acquired immunity apparently wore out and a new boil appeared; after a dose of 250 million cocci a crop of pimples appeared, but soon aborted. Two more abortive boils followed at intervals of three months each. The other element in the treatment on which the author lays stress is the painting of each centre as soon as possible with liquor iodi fortis; by this means many pustules were withered up before they had time to reach the phlegmonous stage of a full-blown boil.

### Adrenalin for the Vomiting of Pregnancy.

Freund has attributed certain phenomena observed in the pregnant woman to insufficiency of the internal secretion of the suprarenals. Two physicians of Modena have attempted to make use of this observation by treating the intractable vomiting of pregnancy with suprarenal extract. Despite their success, they have lacked followers, and the method has fallen into disuse. Dr. R. Robinson has, however, recently reported to the Académie des Sciences two cases in which, after failure of the ordinary methods of treatment, he made use of this extract with excellent results. He employed a 1 in 1000 solution of adrenalin, giving in one case 10 drops a day by the mouth for several days; in the other, the graver case, the same dose by hypodermic injections.

### Poisoning by Vermifuges.

An article from the pen of Dr. Mayor draws attention in *La Semaine Médicale* to the accidents which sometimes follow the administration of santonin and other vermifuges. These he attributes largely to the artifices which are made use of to render the action of the drug more efficacious. He cites the case of a small child rendered blind by a dose of santonin given in oily solution. He points out that the use of sodium santoninate has already been given up in favour of santonin, and declares that oily solutions should be likewise cast aside. As regards extract of male fern, castor oil should not be given as a purgative after its administration, for a case has been reported in which a child died after taking 8 grammes of male fern followed by 30 grammes of castor oil, whereas a fortnight previously the same child had taken with impunity twice that dose of male fern, but followed by a purgative other than castor oil. The same precautions should be taken with regard to santonin as with regard to male fern; no oil of any kind should be used either as a solvent or as a purgative. In conclusion the author sums up by stating that if santonin can cause ocular disturbances such as xanthopsia, its toxicity must not be exaggerated. Care should be taken in giving it, both as to dosage and to the age and susceptibility of the patient. It should be stopped as soon as xanthopsia appears. Its insolubility should be respected, and all attempts to make it soluble, whether in oil or otherwise, avoided.

